# GPT-based chatbot tools are still unreliable in the management of prosthetic joint infections

**DOI:** 10.1007/s12306-024-00846-w

**Published:** 2024-07-02

**Authors:** M. Bortoli, M. Fiore, S. Tedeschi, V. Oliveira, R. Sousa, A. Bruschi, D. A. Campanacci, P. Viale, M. De Paolis, A. Sambri

**Affiliations:** 1 Orthopedic and Traumatology Unit, IRCCS Azienda Ospedaliero-Universitaria Di Bologna, 40138 Bologna, Italy; 2 Department of Medical and Surgical Sciences, Alma Mater Studiorum University of Bologna, 40138 Bologna, Italy; 3 Infectious Disease Unit, Department for Integrated Infectious Risk Management, IRCCS Azienda Ospedaliero-Universitaria Di Bologna, 40138 Bologna, Italy; 4 Department of Orthopedics, Centro Hospitalar Universitário de Santo António, 4099-001 Porto, Portugal; 5 Orthopedic Oncology Unit, Azienda Ospedaliera Universitaria Careggi, 50134 Florence, Italy

**Keywords:** Chatgpt, Artificial intelligence, Orthopedic infection, Periprosthetic joint infection

## Abstract

**Background:**

Artificial intelligence chatbot tools responses might discern patterns and correlations that may elude human observation, leading to more accurate and timely interventions. However, their reliability to answer healthcare-related questions is still debated. This study aimed to assess the performance of the three versions of GPT-based chatbots about prosthetic joint infections (PJI).

**Methods:**

Thirty questions concerning the diagnosis and treatment of hip and knee PJIs, stratified by a priori established difficulty, were generated by a team of experts, and administered to ChatGPT 3.5, BingChat, and ChatGPT 4.0. Responses were rated by three orthopedic surgeons and two infectious diseases physicians using a five-point Likert-like scale with numerical values to quantify the quality of responses. Inter-rater reliability was assessed by interclass correlation statistics.

**Results:**

Responses averaged “good-to-very good” for all chatbots examined, both in diagnosis and treatment, with no significant differences according to the difficulty of the questions. However, BingChat ratings were significantly lower in the treatment setting (*p* = 0.025), particularly in terms of accuracy (*p* = 0.02) and completeness (*p* = 0.004). Agreement in ratings among examiners appeared to be very poor.

**Conclusions:**

On average, the quality of responses is rated positively by experts, but with ratings that frequently may vary widely. This currently suggests that AI chatbot tools are still unreliable in the management of PJI.

## Introduction

Machine learning artificial intelligence (AI) utilizes computational techniques to analyze extensive datasets, categorize information, predict outcomes, or derive valuable insights without explicit instructions [1, 2].

In the contemporary healthcare landscape, the integration of AI has emerged as a transformative force, poised to revolutionize various aspects of medical research and clinical practice [3]. It might discern patterns and correlations that may elude human observation, leading to more accurate and timely interventions.

Within AI applications, natural language processing plays a pivotal role, with language models like ChatGPT, developed by OpenAI (OpenAI LLC, San Francisco, CA, USA), are currently exhibiting remarkable capabilities. ChatGPT, a chatbot (a program able to understand and generate responses using a text-based interface) based on the generative pre-trained transformer (GPT) architecture, uses neural networks to process natural language and produce contextually relevant responses [3].

Recent studies have evaluated ChatGPT performance on general medicine questions and subspecialty training examinations, generally yielding positive results. [4–16]. However, it is crucial to thoroughly assess the knowledge and accuracy of AI models, especially as the complexity of questions and scenarios increases. This evaluation is essential for determining the reliability of AI models in medical decision-making and their feasibility for widespread adoption [17, 18]. Some concerns have been raised about ChatGPT accuracy, precision, legal implications, and references [19]. The phenomenon of “AI hallucination”, where AI generates convincing but entirely fabricated answers, adds complexity to the assessment [19, 20]. Ensuring that ChatGPT and similar AI tools provide trustworthy information is crucial to prevent decisions based on inaccurate or misleading outputs. Additionally, maintaining the integrity of the physician–patient relationship is imperative, as reliance on AI in healthcare settings may unintentionally erode patient trust in healthcare providers. Navigating these challenges with prudence is essential, recognizing the ethical dimensions involved in integrating AI into patient care to mitigate the risk of negative repercussions.

In the context of diseases that require a multidisciplinary approach and intersecting competencies [21], the prospect of AI tools assistance becomes particularly enticing for healthcare professionals in decision-making processes and for patients seeking information along complex diagnostic and therapeutic paths [18, 22]. Prosthetic joint infections (PJI) in orthopedics serve as a compelling example of such scenarios. PJI, a challenging complication after joint replacement, requires prompt and accurate identification for their effective management and to choose the most suitable treatment [23]. While, ChatGPT offers a new way to assist diagnostics through textual data analysis, a thorough understanding of the reliability of different GPT-based chatbot versions in the complex realm of PJI is still to be fully explored. Despite many papers investigated AI chatbots in several orthopedic scenarios, only one recent paper assessed it in PJI field [15].

The aim of this study was to assess the performance of ChatGPT 3.5, BingChat and ChatGPT 4.0 concerning PJI, based on the opinions of a team of experts.

## Materials and methods

The study was conducted in adherence to the ethical standards outlined in the declaration of Helsinki. This study was Institutional Review Board statement exempt as no patient-level or animal-level data were used.

A dataset of 30 questions concerning the diagnosis (15) and treatment (15) of hip and knee PJI was collectively generated by a panel of five physicians from different centers, including three orthopedic surgeons and two infectious disease specialists (ID), all with specific expertise in musculoskeletal infections (Table 1). The physicians engaged were instructed to formulate clear and unambiguous questions. The physicians included were also asked to formulate the questions into three groups by increasing degree of difficulty, according to the collective judgment of all participants. To guarantee consistency, all questions were entered into the examined chatbot versions of the GPT engine by a single researcher. To maintain a systematic approach, mitigating potential bias from AI learning by reinforcement (the ability to incorporate feedback and corrections from human input), data collection and response generation period were limited to a single day by opening new chat sessions within the chatbot interface for each question.

**Table 1:** Questions on diagnosis and treatment of periprosthetic infections used in this study

Domain	Difficulty	Q.	Full-text query
Diagnosis	Easy	1.1.1	What are the typical symptoms of PJI?
		1.1.2	What are the possible causes of PJI?
		1.1.3	What are the diagnostic criteria PJI?
		1.1.4	Would it be useful to perform an arthrocentesis of the affected joint to diagnose PJI?
		1.1.5	What diagnostic laboratory tests are useful for a diagnosis of PJI?
	Medium	1.2.1	Does the presence of a sinus tract communicating to the joint that has previously undergone prosthetic replacement allow the diagnosis PJI?
		1.2.2	Is prosthetic loosening on radiographs a sign of PJI?
		1.2.3	What conditions increase the risk of PJI?
		1.2.4	Are nuclear medicine examinations useful in diagnosing PJI?
		1.2.5	In a patient who underwent joint replacement 2 months ago, does the presence of pain and swelling at the affected joint, fever, serum CRP > 10 mg/L, serum ESR > 30 mm/h, synovial fluid WBC > 3000 cells/µL with > 80% neutrophils, microbiological cultures from synovial fluid positive for S. *epidermidis*, and with an MRI showing a fluid collection into the joint, allow a diagnosis of PJI?
	Hard	1.3.1	Which nuclear medicine method has the highest sensitivity for the diagnosis of PJI?
		1.3.2	Can molecular biology techniques be used to diagnose PJI?
		1.3.3	In a patient who underwent joint replacement 2 months ago, does the presence of pain and swelling at the affected joint, no fever, serum CRP 8 mg/L, serum ESR > 32 mm/h, synovial fluid WBC 1300 cells/µL with 62% neutrophils, allow a diagnosis of PJI? If not, what examinations could be useful?
		1.3.4	What is the role of synovial fluid Leukocyte Esterase and Alpha-Defensin in the diagnosis of PJI?
		1.3.5	When should a PJI with fungal etiology be suspected?
Treatment	Easy	2.1.1	What are the possible surgical treatments of PJI?
		2.1.2	Is intravenous antibiotic therapy alone generally recommended in the treatment of chronic PJI?
		2.1.3	What are the ideal conditions to effectively treat PJI with DAIR?
		2.1.4	What are the ideal conditions to effectively treat PJI with one-stage protocol?
		2.1.5	When a two-stage procedure is most recommended to effectively treat PJI?
	Medium	2.2.1	How can bacterial biofilm maturation on an orthopedic prosthesis influence treatment choice for PJI?
		2.2.2	What is the role of arthroscopic debridement without prosthesis removal in the treatment of PJI?
		2.2.3	When can an articulating cemented spacer be used instead of a static spacer in the two-stage procedure for PJI of TKA?
		2.2.4	When can it be useful to use a mega prosthesis in the treatment of chronic PJI?
		2.2.5	In the case of PJI, how can intraoperative culture investigations and subsequent knowledge of the specific bacterial etiology contribute to the treatment?
	Hard	2.3.1	How can a fungal etiology influence the surgical treatment of PJI?
		2.3.2	In case of severe bone loss after explantation of a THA for PJI, what are the possible reconstruction techniques?
		2.3.3	When should knee arthrodesis be considered as a possible treatment of PJI?
		2.3.4	When should chronic suppressive antibiotic therapy be considered as a possible definitive treatment of PJI?
		2.3.5	In case of periprosthetic knee infection complicated by severe soft tissue injury, with exposure of the prosthesis, what are the possible treatments to consider avoiding above-knee amputation?

PJI, periprosthetic joint infection; WBC, white blood cells count; CRP, C-reactive protein; ESR, erythrocyte sedimentation rate; MRI, magnetic resonance imaging; DAIR, debridement and implant retention; TKA, total knee arthroplasty; THA, total hip arthroplasty

Two different AI applications were examined in this study, OpenAI GPT-3.5 and OpenAI GPT-4. GPT-3.5 and GPT-4 both are language learning machines developed by OpenAI, but they differ in key areas, including model size and computational ability [24]. BingChat, developed by Microsoft (Microsoft Corporation, Redmond, WA, USA) and recently rebranded as Copilot, was also included in this study. BingChat, although powered by OpenAI GPT-4 engine, differs in the mode and style of response reporting (the "balanced" mode was used in this study). The decision to include BingChat in this study was a consequence of the authors’ opinion that, beyond the sources and search technology employed, the response output modality plays a crucial role in users’ evaluation and interpretation of responses.

The responses to each question were provided by ChatGPT 3.5, BingChat, and ChatGPT 4.0, collected by a single researcher not included among the raters, and evaluated individually by each rater. For each question, the three responses provided by the various AI chatbot tools were presented in random order to the raters, hiding the source of the response, in order to blind the experts’ evaluation and avoid the possible bias of judgment resulting from the examiner’s expectations. Prior to the response rating, experts were asked to answer specific questions to define their a priori assumptions about the possible outcome of the analysis, including questions regarding expectations about performance variability depending on the topic of the question, complexity, and GPT version examined.

The evaluation employed a five-point Likert-like scale: 0—insufficient, 5—barely sufficient, 10—good, 15—very good, 20—excellent. The use of numerical values was preferred in order to allow simulated numerical quantification of mean ratings and related quantitative comparisons between groups. To avoid bias, the assessment was conducted in separate settings, guaranteeing that one rater’s judgment did not influence the judgment of another. The evaluation considered various aspects of the provided information, including (1) the focus—defined as the ability to strike the key point of the question, (2) the accuracy—defined as preciseness of details and absence of vague or wrong information, and (3) the completeness—defined as the ability not to neglect relevant information. For each response, a score was assigned according to the Likert-type scale used for each of the three subcriteria (focus, accuracy, completeness). The final response rating was then the sum of the scores for the three subcriteria. The mean ratings of responses for each GPT-based chatbot examined (ChatGPT 3.5, BingChat, ChatGPT 4.0) were compared and further analyzed: (1) by domain (diagnosis and treatment), in order to identify areas of lower and higher reliability of the AI tools; (2) by a priori degree of difficulty, in order to assess the extent of correlation between performance and complexity according to expert assessment.

The degree of inter-rater reliability (IRR) has been also assessed, in order to establish the consistency and the generalizability of the assessment.

## Statistics

Quantitative data were summarized by means and standard deviations. A parametric test was used to compare samples in case of continuous variables and normal distribution, otherwise a nonparametric test was used. The Shapiro–Wilk test was used to verify normal distribution. As a parametric test, the two-tailed Student T-test was used to compare the average of the variables for unpaired groups in case of two groups to be compared and the one-way ANOVA test was used in case of more groups, with post hoc testing using the HSD Tukey test. As a nonparametric test, the two-tailed Mann–Whitney U-test for unpaired groups was used in case of two groups to be compared and the Krusckal-Wallis test was used in case of more groups. Continuity correction was applied due to the discrete distribution. The intraclass correlation coefficient (ICC) was used to quantify the degree of IRR. The assessment of the degree of agreement was performed according to Koo et al. (< 0.50: poor, 0.50 ≤ *x* < 0.75: moderate; 0.75 ≤ *x* < 0.90: good, ≥ 0.90: excellent) [25]. A *p *value < 0.05 was considered statistically significant. All analyses were completed using the Statistical Package for Social Science (IBM Corp. Released 2013. IBM SPSS Statistics for Windows, Version 26.0. Armonk, NY, USA: IBM Corp.).

## Results

The global mean rating, including the responses (no. 450) of all three versions GPT-based chatbots to the 30 expert-generated questions (Table 1), accounting for the ratings of all examiners, was 37.6 ± 1.9, relative to a maximum hypothetical score of 60, thus a summary rating of “good-very good” (as for scores between 30 and 45). The overall mean rating was 38.6 for ChatGPT 3.5, 35.4 for BingChat, 38.7 for ChatGPT 4.0, with no statistically significant differences between versions (*p* = 0.339), even when considering the subcriteria (focus, accuracy, completeness) assessed individually (Table 2).

**Table 2:** Comparison of ratings according to the subcriteria evaluated

Domain	Subcriteria	Mean evaluation			*p* value
		ChatGPT 3.5	BingChat	ChatGPT 4.0	
Diagnosis	Focus	15.0	15.3	14.8	0.908
	Accuracy	10.3	12.4	12.3	0.147
	Completeness	10.9	12.3	12.2	0.513
	Overall	36.2	39.9	39.3	0.498
Treatment	Focus	15.7	13.8	13.9	0.198
	Accuracy	12.0	8.5	12.1	0.020*
	Completeness	13.3	8.5	12.3	0.004*
	Overall	41.1	30.8	38.2	0.025*
Overall	Focus	15.4	14.5	14.3	0.395
	Accuracy	11.2	10.5	12.2	0.199
	Completeness	12.1	10.4	12.2	0.138
	Overall	38.6	35.4	38.7	0.339

*Statistically significant

Regarding the individual domains of question pertinence, there was no significant difference between the global performance of all three versions within the diagnosis field and that within the treatment field (*p* = 0.197). However, the global mean performance in the domain of diagnosis (38.5 ± 2) was higher than that in the domain of treatment (36.7 ± 5.3). This is consistent with the expectations of the examiners, who were interrogated about the hypothetical outcome of the analysis before performing the question ratings. No significant differences were found among the mean ratings of the responses of the three versions, evaluated individually, to the questions about diagnosis, either overall or across subcriteria. In contrast, a lower mean performance of BingChat emerged within the treatment domain compared to the other two versions, both overall (*p* = 0.025) and particularly for the subcriteria “accuracy” (*p* = 0.020) and “completeness” (*p* = 0.004) (Table 2). Regarding the performance of the different versions, the examiners’ expectations were geared in three out of five cases toward no difference between the versions and in two out of five cases toward a better performance of ChatGPT 4.0 than the other two versions.

No significant differences were found between the mean ratings of responses to the questions stratified by a priori established complexity, either overall or within individual domains of pertinence (Table 3). This was observed both when considering the overall mean ratings of the three versions and when comparing the performance of the individual versions examined (Table 3).

**Table 3:** Comparison of ratings according to the complexity of the queries

Domain	Difficulty	Mean evaluation			p-value
		ChatGPT 3.5	BingChat	ChatGPT 4.0	
Diagnosis	Easy	37.2	37.6	40.8	0.681
	Medium	32.6	46.6	41.6	0.859
	Hard	38.8	35.6	35.4	0.855
	*p* value	0.592	0.164	0.472	–
Treatment	Easy	36.2	29.4	42.2	0.159
	Medium	43.8	33.4	35.4	0.345
	Hard	43.2	29.6	37.0	0.167
	*p* value	0.462	0.799	0.598	–
Overall	Easy	36.7	33.5	41.5	0.120
	Medium	38.2	40.0	38.5	0.927
	Hard	41.0	32.6	36.2	0.206
	*p* value	0.672	0.249	0.458	–

As regards inter-rater reliability (IRR) among examiners, this was found to be consistently poor (ICC < 0.5) [25], both in general and within individual domains of question pertinence (Table 4). A subgroup analysis was conducted both according to examiners’ years of experience and their specialization. A similar result also emerged from stratification by specialization, with the infectiologists’ IRR higher than that of orthopedic surgeons.

**Table 4:** Inter-rater reliability (IRR) expressed by intraclass correlation coefficient (ICC)

Domain	Subcriteria	IRR via ICC						
		< seven years experience	> seven years experience	*p* value	Orthopedic surgeons	Infectivologists	*p* value	Overall
Diagnosis	Focus	–	–	–	–	–	–	0.22
	Accuracy	–	–	–	–	–	–	0.03
	Completeness	–	–	–	–	–	–	0.05
	Overall	0.29	0.10	< 0.001*	0.13	0.23	0.002*	0.20
Treatment	Focus	–	–	–	–	–	–	0.06
	Accuracy	–	–	–	–	–	–	0.10
	Completeness	–	–	–	–	–	–	0.01
	Overall	0.20	0.07	< 0.001*	0.15	0.17	0.691	0.18
Overall	Focus	–	–	–	–	–	–	0.15
	Accuracy	–	–	–	–	–	–	0.17
	Completeness	–	–	–	–	–	–	0.03
	Overall	0.25	0.09	< 0.001*	0.14	0.21	0.026*	0.19

*Statistically significant

## Discussion

The treatment of PJI represents a real challenge for patients, healthcare providers and the healthcare system itself, because of the high number of treatment failures and the high economic burden of these diseases [21, 26]. While AI, exemplified by ChatGPT, has a significant potential to assist PJI management by healthcare professionals and to simplify information retrieval for patients, the reliability of these technologies in a medical setting requires rigorous examination. This study attempted to address some of the existing gaps by subjecting various iterations of ChatGPT to thorough evaluation by a diverse panel of experts, consisting of orthopedic surgeons and infectious disease physicians.

As for the present study, a significant aspect arose is the substantial consistency of the average performance among the different versions of the language models. Despite variations in architectures and implementations, the results demonstrated good overall efficiency, thus suggesting inherent robustness.

The main finding of the present study is the absence of any statistically significant differences were observed in the average ratings either among the three subcriteria analyzed in the responses (focus, accuracy, completeness) or according to the domain of pertinence of the questions (diagnosis or treatment). However, regarding the latter point, it should be noted that the average ratings of treatment-related responses were slightly lower, in line with the results of studies by Draschl et al. [15] and Lum et al. [27], who had already detected this trend. This may be attributed to the less standardization of the treatment of PJI compared with diagnostics, with less univocal and standardized criteria for choosing among treatments. In addition, this could be due to weaker modeling and poor utilization of relevant orthopedic literature in the ChatGPT pretraining stage [10]. Several previous studies have shown that the performance of the ChatGPT tends to decrease with increasing question complexity [11, 28]. Interestingly, the responses of all the AI chatbots analyzed in this study to questions Q1.2.5 and Q1.3.3—which posed clinical questions about the ability to diagnose PJI in specific cases with increasing complexity and numerous variables—were all rated positively on average. In these cases, the explanation could be related to the existence of clear yes/no diagnostic criteria that the AI is allowed to refer to. In the authors’ opinion, the concept of “complexity” for AI should not be purely equated with its definition related to human intelligence. It should be rather construed not only in terms of the number of variables to be analyzed and their relationships, but also by considering the possibility of access to clear and discriminating sources that can reduce the need for critical thinking, an evident gap in AI. Even within the same domain (diagnosis or treatment), no significant differences in performance were observed in relation to query complexity. This could be ascribed to the different meaning attributed to the concept of complexity for human and artificial intelligence, which would require the definition of objective and standardized criteria for proper evaluation. Finally, it is noteworthy that in the analysis of the average performance of the different versions, no differences emerged between ChatGPT 3.5 and 4.0 (in contrast to the study in the orthopedic field by Massey et al. [10]), while BingChat exhibited significantly lower results than the other models on treatment questions. This discrepancy could be attributed to the specific answer reporting methods implemented by BingChat, despite its operation being based on GPT-4.

Another aspect to consider is the finding of a low inter-rater reliability (IRR) score, a result that deviates from expectations and previous studies [12, 29, 30], including a recent study on ChatGPT reliability in the context of PJI [15]. It can be speculated that the nature of the questions employed, which primarily required textual responses not limited to the yes/no format, may have been more susceptible to different individual interpretations. It is also possible that the larger number of examiners who were recruited in this study contributed to this difference, along with potential differences in raters’ characteristics. The implications of a low IRR are crucial in understanding the reliability and generability of the results found [22, 28, 31, 32]. The questions selected by physicians in the present study and in similar studies were designed to be clear and lead to uncontroversial answers, possibly including instructions for incorporating the guidelines, and thus are probably not representative of the questions that patients and the general public, who have no explicit knowledge to incorporate, would generate. ChatGPT has demonstrated a high level of applied medical and clinical knowledge and it may be expected to acquire a greater role in patient care in the future. However, the human factor, critical thinking and clinical judgment remain imperative to ensure the accuracy and safety of medical diagnosis and treatment decisions. GPT-based chatbot tools are currently arguably suitable for a complementary role [4]. In fact, these models have the potential to be used to provide additional support in the interpretation of clinical data by healthcare professionals, and they also are amenable to use by patients, but with supervision or at least the opportunity for discussion with referring physicians.

In the future, it might be interesting to replicate this type of study to investigate patients’ perspective by asking them to evaluate the answers provided to their questions by both experts and ChatGPT. This would allow to examine not only the performance of GPT-based chatbots toward less accurately formulated questions, but also their communicative effectiveness compared to that of physicians.

This study has several limitations. As discussed, the study relied on the subjective and self-reported evaluations of physicians, which may introduce bias; similar judgments may also vary from one physician to another, depending on individual characteristics such as experience, habits, and area of expertise. Experts may be influenced by past expectations, introducing an additional subjective element in the evaluation of AI performance. The number of evaluators was low, as was the total number of questions, although this is in line with the purpose of the study to establish the scope of the evaluation in a well-defined context. The proposed questions were generated by a team of experts but lacked standardized criteria for stratifying complexity. These elements may have affected the performance of the AI chatbots examined, partially altering the quality of comparative considerations. Also, it is critical to note that ChatGPT is an evolving AI network that continuously learns and improves over time, limiting the reproducibility of the study, even if the same methodology is used. A further limitation of this study is not having included other versions of AI chatbots currently available, particularly the more recently released Bard AI, developed by Google (Google LLC, Mountain View, CA, USA) which would be worthy of investigation in future studies.

Exploring the potential of AI chatbot tools in medical applications requires a concerted effort to address existing uncertainties and challenges. By adopting a collaborative and standardized approach, it will be possible to contribute to building a solid evidence base, laying the foundation for responsible and effective integration of AI technologies in the orthopedic practice and, by extension, in healthcare at large.

## Conclusions

This analysis of the performance of different versions of GPT-based chatbot tools in the context of periprosthetic joint infections found high average quality of expert evaluations of the responses. However, the low agreement of ratings, although not clearly explained, poses an interesting challenge for future research by highlighting the complexity of subjective evaluation in textual responses. This suggest that the use of AI chatbot tools is still unreliable in the management of PJI. The potential for greater future integration of these models into clinical practice is undeniable, but medical professionals and patients should be aware of the limitations and actively verify AI-generated medical information with trusted sources. Currently, increasing the number of available studies and standardizing their methodologies remains perhaps the best means of acquiring robust evidence.

## Data Availability

Data sharing is not applicable to this article.

## References

[1] Helm JM, Swiergosz AM, Haeberle HS, Karnuta JM, Schaffer JL, Krebs VE, Spitzer AI, Ramkumar PN (2020) Machine learning and artificial intelligence: definitions, applications, and future directions. Curr Rev Musculoskelet Med 13:69–76. 10.1007/s12178-020-09600-8 31983042 10.1007/s12178-020-09600-8 PMC7083992

[2] Jordan MI, Mitchell TM (2015) Machine learning: trends, perspectives, and prospects. Science 349:255–260. 10.1126/science.aaa8415 26185243 10.1126/science.aaa8415

[3] Sallam M (2023) ChatGPT utility in healthcare education, research, and practice: systematic review on the promising perspectives and valid concerns. Healthcare. 10.3390/healthcare11060887 36981544 10.3390/healthcare11060887 PMC10048148

[4] AlessandriBonetti M, Giorgino R, GalloAfflitto G, De Lorenzi F, Egro FM (2023) How does ChatGPT perform on the Italian residency admission national exam compared to 15,869 medical graduates? Ann Biomed Eng. 10.1007/s10439-023-03318-7 37490183 10.1007/s10439-023-03318-7

[5] Gilson A, Safranek CW, Huang T, Socrates V, Chi L, Taylor RA, Chartash D (2023) How does ChatGPT perform on the United States medical licensing examination? The implications of large language models for medical education and knowledge assessment. JMIR Med Educ 9:e45312. 10.2196/45312 36753318 10.2196/45312 PMC9947764

[6] Hoch CC, Wollenberg B, Luers JC, Knoedler S, Knoedler L, Frank K, Cotofana S, Alfertshofer M (2023) ChatGPT’s quiz skills in different otolaryngology subspecialties: an analysis of 2576 single-choice and multiple-choice board certification preparation questions. Eur Arch Otorhinolaryngol 280:4271–4278. 10.1007/s00405-023-08051-4 37285018 10.1007/s00405-023-08051-4 PMC10382366

[7] Jung LB, Gudera JA, Wiegand TLT, Allmendinger S, Dimitriadis K, Koerte IK (2023) ChatGPT passes German state examination in medicine with picture questions omitted. Dtsch Arztebl Int 120:373–374. 10.3238/arztebl.m2023.0113 37530052 10.3238/arztebl.m2023.0113 PMC10413971

[8] Kung JE, Marshall C, Gauthier C, Gonzalez TA, Jackson JB (2023) Evaluating ChatGPT performance on the orthopaedic in-training examination. JB JS Open Access. 10.2106/JBJS.OA.23.00056 37693092 10.2106/JBJS.OA.23.00056 PMC10484364

[9] Kung TH, Cheatham M, Medenilla A, Sillos C, De Leon L, Elepano C, Madriaga M, Aggabao R, Diaz-Candido G, Maningo J et al (2023) Performance of ChatGPT on USMLE: potential for AI-assisted medical education using large language models. PLOS Digit Health 2:e0000198. 10.1371/journal.pdig.0000198 36812645 10.1371/journal.pdig.0000198 PMC9931230

[10] Massey PA, Montgomery C, Zhang AS (2023) Comparison of ChatGPT-3.5, ChatGPT-4, and orthopaedic resident performance on orthopaedic assessment examinations. J Am Acad Orthop Surg 31:1173–1179. 10.5435/JAAOS-D-23-00396 37671415 10.5435/JAAOS-D-23-00396 PMC10627532

[11] Moshirfar M, Altaf AW, Stoakes IM, Tuttle JJ, Hoopes PC (2023) Artificial intelligence in ophthalmology: a comparative analysis of GPT-35, GPT-4, and human expertise in answering StatPearls questions. Cureus 15:e40822. 10.7759/cureus.40822 37485215 10.7759/cureus.40822 PMC10362981

[12] Passby L, Jenko N, Wernham A (2023) Performance of ChatGPT on dermatology specialty certificate examination multiple choice questions. Clin Exp Dermatol. 10.1093/ced/llad197 37264670 10.1093/ced/llad197

[13] Saad A, Iyengar KP, Kurisunkal V, Botchu R (2023) Assessing ChatGPT’s ability to pass the FRCS orthopaedic part A exam: a critical analysis. Surgeon 21:263–266. 10.1016/j.surge.2023.07.001 37517980 10.1016/j.surge.2023.07.001

[14] Takagi S, Watari T, Erabi A, Sakaguchi K (2023) Performance of GPT-3.5 and GPT-4 on the Japanese medical licensing examination: comparison study. JMIR Med Educ 9:e48002. 10.2196/48002 37384388 10.2196/48002 PMC10365615

[15] Draschl A, Hauer G, Fischerauer SF, Kogler A, Leitner L, Andreou D, Leithner A, Sadoghi P (2023) Are ChatGPT’s free-text responses on periprosthetic joint infections of the hip and knee reliable and useful? J Clin Med. 10.3390/jcm12206655 37892793 10.3390/jcm12206655 PMC10607052

[16] Uz C, Umay E (2023) “Dr ChatGPT”: is it a reliable and useful source for common rheumatic diseases? Int J Rheum Dis 26:1343–1349. 10.1111/1756-185X.14749 37218530 10.1111/1756-185X.14749

[17] O’Connor S (2023) Open artificial intelligence platforms in nursing education: tools for academic progress or abuse? Nurse Educ Pract 66:103537. 10.1016/j.nepr.2022.103537 36549229 10.1016/j.nepr.2022.103537

[18] Liu S, Wright AP, Patterson BL, Wanderer JP, Turer RW, Nelson SD, McCoy AB, Sittig DF, Wright A (2023) Using AI-generated suggestions from ChatGPT to optimize clinical decision support. J Am Med Inform Assoc 30:1237–1245. 10.1093/jamia/ocad072 37087108 10.1093/jamia/ocad072 PMC10280357

[19] Shen Y, Heacock L, Elias J, Hentel KD, Reig B, Shih G, Moy L (2023) ChatGPT and other large language models are double-edged swords. Radiology 307:e230163. 10.1148/radiol.230163 36700838 10.1148/radiol.230163

[20] Athaluri SA, Manthena SV, Kesapragada V, Yarlagadda V, Dave T, Duddumpudi RTS (2023) Exploring the boundaries of reality: investigating the phenomenon of artificial intelligence hallucination in scientific writing through ChatGPT references. Cureus 15:e37432. 10.7759/cureus.37432 37182055 10.7759/cureus.37432 PMC10173677

[21] Sambri A, Fiore M, Tedeschi S, De Paolis M (2022) The Need for multidisciplinarity in modern medicine: an insight into orthopaedic infections. Microorganisms 10:756 35456807 10.3390/microorganisms10040756 PMC9028939

[22] Bernstein J (2023) Not the last word: ChatGPT can’t perform orthopaedic surgery. Clin Orthop Relat Res 481:651–655. 10.1097/CORR.0000000000002619 36877168 10.1097/CORR.0000000000002619 PMC10013616

[23] Parvizi J, Tan TL, Goswami K, Higuera C, Della Valle C, Chen AF, Shohat N (2018) The 2018 definition of periprosthetic hip and knee infection: an evidence-based and validated criteria. J Arthroplasty 33:1309–1314. 10.1016/j.arth.2018.02.078 29551303 10.1016/j.arth.2018.02.078

[24] Achiam J, Adler S, Agarwal S, Ahmad L, Akkaya I, Aleman FL, Almeida D, Altenschmidt J, Altman S, Anadkat S et al. (2023) GPT-4 Technical Report. https://cdn.openai.com/papers/gpt-4.pdf. Aaccessed 20 Oct 2023

[25] Koo TK, Li MY (2016) a guideline of selecting and reporting intraclass correlation coefficients for reliability research. J Chiropr Med 15:155–163. 10.1016/j.jcm.2016.02.012 27330520 10.1016/j.jcm.2016.02.012 PMC4913118

[26] Zardi EM, Franceschi F (2020) Prosthetic joint infection. A relevant public health issue. J Infect Public Health 13:1888–1891. 10.1016/j.jiph.2020.09.006 33289642 10.1016/j.jiph.2020.09.006

[27] Lum ZC (2023) Can artificial intelligence pass the American board of orthopaedic surgery examination? Orthopaedic residents versus ChatGPT. Clin Orthop Relat Res 481:1623–1630. 10.1097/CORR.0000000000002704 37220190 10.1097/CORR.0000000000002704 PMC10344569

[28] Johnson D, Goodman R, Patrinely J, Stone C, Zimmerman E, Donald R, Chang S, Berkowitz S, Finn A, Jahangir E et al (2023) Assessing the accuracy and reliability of AI-generated medical responses: an evaluation of the Chat-GPT model. Res Sq. 10.21203/rs.3.rs-2566942/v1 39108497 10.21203/rs.3.rs-3417165/v1 PMC11302681

[29] Zalzal HG, Cheng J, Shah RK (2023) Evaluating the current ability of ChatGPT to assist in professional otolaryngology education. OTO Open 7:e94. 10.1002/oto2.94 38020045 10.1002/oto2.94 PMC10663981

[30] Goodman RS, Patrinely JR, Stone CA Jr, Zimmerman E, Donald RR, Chang SS, Berkowitz ST, Finn AP, Jahangir E, Scoville EA et al (2023) Accuracy and reliability of chatbot responses to physician questions. JAMA Netw Open 6:e2336483. 10.1001/jamanetworkopen.2023.36483 37782499 10.1001/jamanetworkopen.2023.36483 PMC10546234

[31] Churchill J, Menendez ME, Ponce BA (2016) Early postoperative complications after shoulder arthroplasty in patients with epilepsy. Orthopedics 39:e1075–e1079. 10.3928/01477447-20160714-02 27458894 10.3928/01477447-20160714-02

[32] Elmahdy M, Sebro R (2023) A snapshot of artificial intelligence research 2019–2021: is it replacing or assisting physicians? J Am Med Inform Assoc 30:1552–1557. 10.1093/jamia/ocad094 37279884 10.1093/jamia/ocad094 PMC10436151

